# Features of Rhabdomyolysis Secondary to Immobility and Statin-Induced Myopathy in a 70-Year-Old Female

**DOI:** 10.7759/cureus.8330

**Published:** 2020-05-28

**Authors:** Arzoo Shahid, Mobassir A Akbar, Madiha Ariff

**Affiliations:** 1 Internal Medicine, University of Alberta Hospital Edmonton, Alberta, CAN; 2 Family Medicine, University of Alberta Hospital Edmonton, Alberta, CAN; 3 Ears, Nose and Throat, Jinnah Post Graduate Medical Center, Karachi, PAK; 4 Internal Medicine, Dow University of Health Sciences, Karachi, PAK

**Keywords:** statin-induced myopathy, rhabdomyolysis, creatine kinase

## Abstract

Treatment with statins requires close monitoring of serum creatine kinase levels to prevent myopathy, which is a rare but potentially serious dose-dependent adverse effect of these drugs. Statins are cholesterol-lowering drugs that are among the most prescribed drugs worldwide and are considered effective in reducing the risk of major cardiovascular events. Although statins are generally well-tolerated, myopathies are a rare but known adverse event, ranging from muscle pain to very rare cases of life-threatening rhabdomyolysis. In this report, we aim to highlight the features of rhabdomyolysis secondary to immobility and statin-induced myopathy.

## Introduction

Although statins, a class of cholesterol-lowering drugs, are generally well-tolerated, myopathy is a rare but known adverse event. It may range from muscle pain to life-threatening rhabdomyolysis in up to 25% of patients [1]. This statin-induced myopathy has been attributed to the type, metabolism, dose, drug-drug interaction, and lipophilicity of the statin used [2]. Several other factors such as age, presence of co-morbid conditions, genetics, gender, and ethnicity of the patient have also been implicated [3]. The development of myopathy in these cases is considered secondary to massive muscle destruction and myoglobinuria, which is indicated by a rise in levels of serum creatine kinase (CK). In around 5% of statin users, the elevation of serum CK has been observed, usually being 2-10 times the upper limit of normal levels [4]. Thus, treatment with statins requires close monitoring of serum CK levels to prevent myopathy. With the stoppage of statin, serum CK levels typically normalize in a matter of few weeks to several months.

## Case presentation

**Figure 1:**
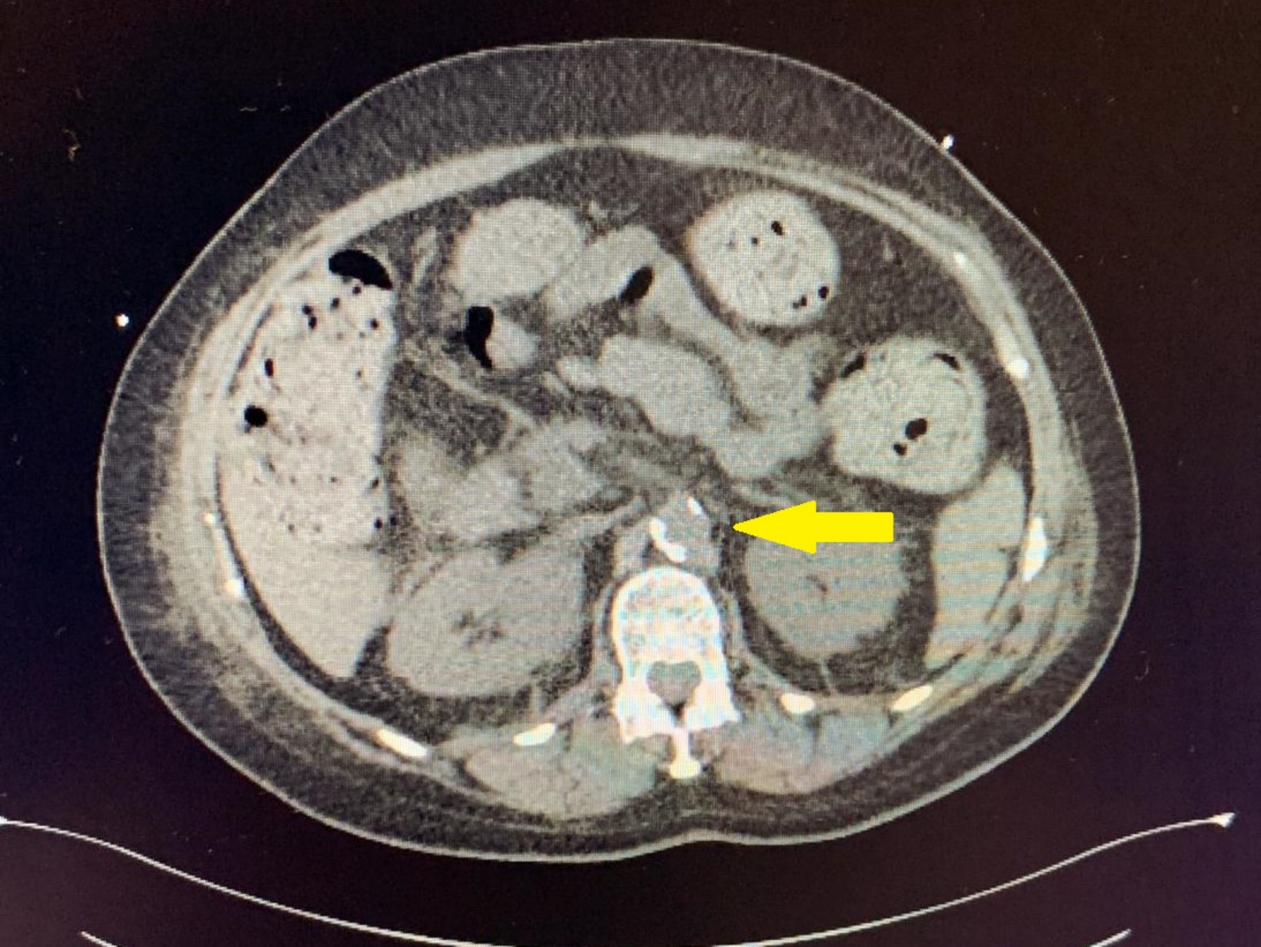
CT scan of the abdomen showing a pancreatic head lesion.

We present a case of statin-induced rhabdomyolysis in a 70-year-old female who presented to the Internal Medicine Department at the University of Alberta Hospital, Edmonton, Canada. The patient had co-morbidities such as congestive heart failure, hypertension, dyslipidemia, severe mitral valve regurgitation, and chronic back pain. She was treated for Hodgkin's lymphoma in 2011 with 12 cycles of chemotherapy, currently in remission. For her dyslipidemia, she was on simvastatin 10 mg once daily (OD) from June 25, 2019, to November 24, 2019. Her general practitioner changed her to rosuvastatin 40 mg OD on November 25, 2019, for a better outcome. She was admitted on January 5, 2020, with complaints of progressive weakness with recurrent falls over the past two to three weeks, along with difficulty in standing. She denied any history of fever, rashes, joint pain, or weakness in hands. On examination, her blood pressure was 123/67 mm Hg, pulse was 95 beats/minute, temperature was 36.4°C, and oxygen saturation was 100% on room air. Her heart sounds were normal with displaced apex, and no murmurs were present. The chest had bibasilar crepitation. The abdomen was soft and non-tender, and no organomegaly was found. Raynaud's phenomenon of the right index finger was found. No obvious nail bed abnormalities, shawl sign, heliotrope rash, or holster sign were present. Anasarca with edema up to the abdomen was found. On musculoskeletal examination, her shoulder power was 4/5 bilaterally, finger flexion and dorsiflexion were 5/5, but hip flexion was 2/5 bilaterally. No hypothenar wasting or fasciculation was seen, and no rash was found. On admission, her hemoglobin was 82 gm/L, WBC was 7.1 x 109/L, platelets were 170 x 109/L, and CK was 47,524 U/L (normal: <190 U/L). She was on a statin that was started eight months ago and was changed from simvastatin to rosuvastatin about three months ago. Elevated CK was attributed initially to rhabdomyolysis secondary to immobility as the patient was bedbound at home for several days, but her CK continued to rise, and that is why her rosuvastatin was held on the second day of her admission. She has initially advised intravenous (IV) fluids that led to a decrease in her CK to 29,592U/L in two days. However, it rose again to 43,000U/L the next day, and as a result the Rheumatology Department was consulted. Her CT scan of the abdomen and pelvis was also performed, which revealed findings suggestive of peritoneal disease, and a primary lesion was suspected to be in the anterior pancreatic head (Figure 1).

**Figure 2:**
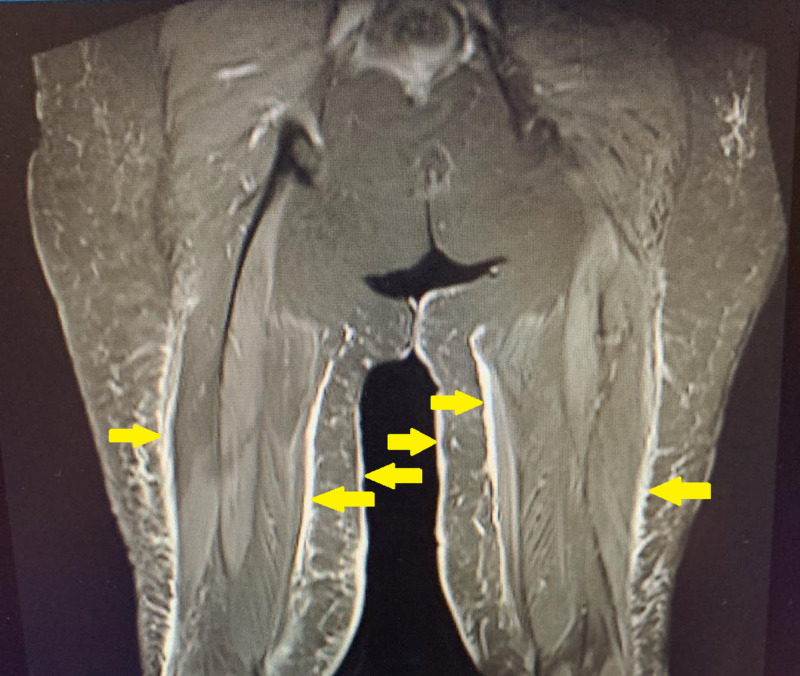
MRI of the hip and thigh showing multi focal myositis of the adductor longus.

MRI of the hips and thighs were also advised by the rheumatologists, which showed multifocal myositis involving mainly bilateral rectus abdominis, adductor longus, sartorius, and semitendinosus muscles, along with diffuse fascial edema in bilateral lower extremities (Figure 2).

Based on history, examination, and investigations, a broad spectrum of differential diagnosis was considered. Top differentials included statin-induced myopathy versus necrotizing autoimmune myositis versus dermatomyositis secondary to malignancy. Some physical examination findings were suggestive of an autoimmune myopathy such as Raynaud's disease. CT of the abdomen with a probable pancreatic head neoplasm hinted toward the diagnosis of paraneoplastic dermatomyositis. Statin therapy was also changed around the time of onset of symptoms, which made it suspicious, and it was therefore discontinued. The rheumatologist advised IV fluids, and IV methylprednisolone for four days, which was later switched to oral prednisone 50 mg/day. Given that patient was put on steroids, Pneumocystis jiroveci pneumonia prophylaxis was started with Septra (trimethoprim and sulfamethoxazole). Muscle biopsy along with autoimmune myositis panel was performed to rule out necrotizing autoimmune myositis. The neurology department was consulted for muscle weakness, and they advised electromyography and nerve conduction studies. The physiotherapist was also involved to improve muscle strength.

Few days after admission, the patient had a fall during her hospital stay, and the next day her CK rose to 82,000 U/L. This was most likely secondary to her fall but might have represented the worsening of her myositis. IV fluids were continued, and oral steroids were also started. CK was closely monitored, and the initial plan was to give IV immunoglobulin for suspected myositis. But a few days later, her CK and creatinine levels came close to normal. The patient was discharged on a tapering dose of steroids, on January 20, 2020, after 15 days of hospital stay. Her investigations including myositis panel, electromyography, and nerve conduction studies came out to be normal. Muscle biopsy, which was initially planned, was later canceled.

## Discussion

Following the findings in our study, where statin use was associated with myopathy leading to rhabdomyolysis, other researchers have also reported that in up to 25% of patients on statins, myopathy causing rhabdomyolysis develops [1]. Even though statins are usually well-tolerated and adverse events are uncommon, the risk of statin-induced myopathy has been attributed to the type of statin used, where differences among them might be explained to a certain extent by each statin’s relative potencies in inhibiting the synthesis of cholesterol in addition to their dependency on other factors such as metabolism, dose, drug-drug interaction, and lipophilicity [2]. Furthermore, several factors attributed to myopathy are related to the patients, as well as age, co-morbidity, genetics, gender, and ethnicity [3]. The link between statin-induced myopathy and multiple genetic polymorphisms includes transportation of a drug, pathway of clearance, and the enzymes involved in the synthesis of creatine [5]. Nonetheless, not every patient who is affected carries such polymorphisms. Studies have also demonstrated an effect of statin-induced myopathy on the disturbances in homeostasis of calcium, reduced cholesterol synthesis at the cellular level, and an increase in expression of HMG-CoA reductase inhibitors [6]. Another attribution of statin-induced myopathy is the part played by mitochondria, where a dysfunctional or decreased coenzyme-Q10 synthesis might play a role [7]. On the other hand, trials on administrating coenzyme-Q10 have reported equivocal results, questioning its pathophysiological role in statin-induced myopathy [8].

In around 5% of users of statins, CK elevation is observed, usually being 2-10 times the upper limit of normal levels, which was similar to the levels reported in our study [4]. Typically, with the stoppage of statin, CK levels normalize, but it may take weeks to months. Similarly, in our study, CK levels drastically dropped when statin use was stopped. Rarely statins lead to a life-threatening, immune-mediated necrotizing myopathy. Diversification of genetics seems to result in differing responses to statin therapy, as reported in a SEARCH (Study of the Effectiveness of Additional Reductions in Cholesterol and Homocysteine) Collaborative Group, a genome-wide study of 300,000 markers among 85 patients diagnosed with statin-induced myopathy and 90 controls, all being on a daily statin. The study found out a single-nucleotide polymorphism gene present on chromosome number 12, which reported to be strongly linked with a greater risk of statin-induced myopathy [9]. Another study observed statin-induced myopathy with CK elevations, proposing a mechanism in which statins may cause an increase in membrane fragility of the muscles due to reduced synthesis of cholesterol content by inhibiting the production of the lipid content of cell membranes, leading to depleted ubiquinone's, and proteins, causing mitochondrial dysfunction [10]. In addition to the lipid-lowering effects, statins also play a role in increasing the stability of atherosclerotic plaques, reducing inflammation and oxidative stress, and improving endothelial function. However, it has been reported in some studies that in around 5% of patients, statin use causes myopathy, with increases in CK levels causing rhabdomyolysis due to massive muscle destruction and myoglobinuria [11]. In yet another research, it has been proposed that very high levels of lipids at baseline indicate the possibility of high statin dose for controlling lipids, thereby contributing to the development of elevations in CK levels, leading to myopathy and rhabdomyolysis [12].

## Conclusions

Though overall patient findings were most consistent with rhabdomyolysis secondary to immobility and statin-induced myopathy, one should always think of a broader differential diagnosis. In such a scenario, top differentials should include statin-induced myopathy, autoimmune myositis, and dermatomyositis secondary to malignancy. Fortunately, in our patient, all of the other investigations came back to normal.
